# Impact of norepinephrine on immunity and oxidative metabolism in sepsis

**DOI:** 10.3389/fimmu.2023.1271098

**Published:** 2023-11-07

**Authors:** Joby Thoppil, Prayag Mehta, Brett Bartels, Drashya Sharma, J. David Farrar

**Affiliations:** ^1^ Department of Emergency Medicine, University of Texas, Southwestern Medical Center, Dallas, TX, United States; ^2^ Department of Immunology, University of Texas (UT) Southwestern Medical Center, Dallas, TX, United States

**Keywords:** norepinephrine, oxidative metabolism, sepsis, sympathetic nervous system, immune cells

## Abstract

Sepsis is a major health problem in the United States (US), constituting a leading contributor to mortality among critically ill patients. Despite advances in treatment the underlying pathophysiology of sepsis remains elusive. Reactive oxygen species (ROS) have a significant role in antimicrobial host defense and inflammation and its dysregulation leads to maladaptive responses because of excessive inflammation. There is growing evidence for crosstalk between the central nervous system and the immune system in response to infection. The hypothalamic-pituitary and adrenal axis and the sympathetic nervous system are the two major pathways that mediate this interaction. Epinephrine (Epi) and norepinephrine (NE), respectively are the effectors of these interactions. Upon stimulation, NE is released from sympathetic nerve terminals locally within lymphoid organs and activate adrenoreceptors expressed on immune cells. Similarly, epinephrine secreted from the adrenal gland which is released systemically also exerts influence on immune cells. However, understanding the specific impact of neuroimmunity is still in its infancy. In this review, we focus on the sympathetic nervous system, specifically the role the neurotransmitter norepinephrine has on immune cells. Norepinephrine has been shown to modulate immune cell responses leading to increased anti-inflammatory and blunting of pro-inflammatory effects. Furthermore, there is evidence to suggest that norepinephrine is involved in regulating oxidative metabolism in immune cells. This review attempts to summarize the known effects of norepinephrine on immune cell response and oxidative metabolism in response to infection.

## Introduction

Sepsis ranks as the tenth leading contributor of mortality in the United States and stands as the second most prevalent cause of death among patients in the Intensive Care Unit (ICU) (1). Sepsis frequently leads to mortality, with rates ranging from 20 to 50 percent worldwide due to refractory multiple organ dysfunction (1–4). Although sepsis is fundamentally thought to be an inflammatory disease, anti-inflammatory therapies have been unsuccessful at reducing mortality. Consequently, current treatment guidelines focus on early identification and intervention. The nervous and immune systems are intrinsically related (5). Immune system molecules and cells cross the blood–brain barrier and send signals which allow for central input. The binding of soluble immune signaling molecules to receptors expressed on the surface of various types of central nervous system (CNS) cells produce cellular responses that direct the sympathetic nervous system (SNS) to secrete norepinephrine (NE) (5). The CNS via the SNS can also induce the adrenal gland to secrete epinephrine systemically. Accumulated evidence over the past two decades suggests that NE acts as a neurotransmitter/neuromodulator within primary and secondary lymphoid organs (6). Therefore, once activated sympathetic nerve terminals release NE which directly act on adrenoreceptors expressed on immune cells within these organs. Similarly, circulating epinephrine released by the adrenal gland can also act on adrenoreceptors (6). Neurons also possess receptors for cytokines and chemokines secreted by the immune system allowing for fine tuning of the local immune response (7).

It has long been established that endotoxin released from bacterial cell walls induces SNS activity (7, 8). Early investigations identified alterations in the level of sympathetic nerve activity by assessing circulating levels of NE and Epi in times of infection and shock. These studies identified consistently that endotoxin exposure led to increased levels of circulating NE, suggesting enhanced sympathetic nerve activity (8). Therefore, immune cell activation following peripheral infectious challenges likely increases the level of CNS mediated regulatory mechanisms (8). NE acts by interacting with adrenergic receptors (ADRs) which are expressed on the surfaces of the cells of the immune system. In human leukocytes, β‐ADRs are expressed on natural killer (NK) cells, monocytes, B cells, CD8^+^ T cells, and CD4^+^ T cells (9–12). Studies have also confirmed that β‐ADR signaling can regulate various functions in the immune system (9–12). For example, in response to NE B‐cell costimulatory molecules and IgE secretion is increased; in monocytes and macrophages NE signaling results in decreased proinflammatory cytokine production; in T cells, Th1 cytokine production by CD4^+^ T cells is decreased, and regulatory T‐cell function is enhanced as a result of NE (9–12). Moreover, there is indication that the SNS can regulate the migration of stem cells from the bone marrow to their designated niche (13). This review will focus on direct noradrenergic (NE) innervation on immune tissue via the SNS.

## The effect of NE on immune cells

NE has been demonstrated to have an overall anti-inflammatory effect, mediated primarily through β‐ADRs. Post-ganglionic sympathetic nerve fibers, which release NE as their primary neurotransmitter, intricately innervate primary and secondary lymphoid tissues (6, 14). Immune cells establish direct contact with the dendrites of these neurons. Most notably, both innate and adaptive immune cells express adrenergic receptors, predominantly the β2-adrenergic receptor (β2‐ADR) allowing them to directly engage with the SNS (6, 14). *In-vitro* studies have shown many anti-inflammatory immunologic effects, including decreased pro-inflammatory tumor necrosis factor alpha (TNF-alpha), Interleukin (IL)-6, and IL-8, and stimulation of anti-inflammatory cytokine IL-10. Many of norepinephrine’s effects have been shown to be dose dependent (15). In macrophages, beta-adrenergic stimulation increases cAMP and inhibits nuclear factor kappa-light-chain-enhancer of activated B cells (NF-κB) from entering the nucleus, reducing pro-inflammatory cytokine transcription, as well as increased production of anti-inflammatory IL-10 (16). IL-10 was shown to markedly inhibit endotoxin-induced TNF-alpha production by mouse and rat macrophages *in vitro* (17). The norepinephrine-induced stimulation of IL-10, in addition to attenuation of TNF-alpha and IL-6, have been shown to be diminished by beta-blockade with medications such as metoprolol and propranolol (15). In other *in vitro* studies, NE diminishes NK cell cytotoxicity in a dose-dependent manner and downregulates IL-2 production through β2‐ADR modulation. These effects were mitigated by administration of propranolol but not by atenolol, indicating an effect mediated by the β2‐ADR.

Deletion of β2‐ADRs in macrophages and dendritic cells have led to significant attenuation of IL-10 and increased TNF-alpha in response to lipopolysaccharide (LPS) administration (9, 18). *In-vivo* sepsis models showed increased mortality with β2‐ADR deletion (9, 18). As part of the neuroinflammatory reflex, vagal nerve stimulation induces NE release from the spleen, resulting in acetylcholine secretion by CD4+ T-cells. Modulation of alpha-7 cholinergic receptors on macrophages by acetylcholine leads to suppression of pro-inflammatory cytokines (19). In animal models, stimulation of the vagal nerve attenuates systemic inflammation, whereas interruption of this cascade by vagotomy increased susceptibility to septic shock secondary to endotoxin effects (19). In patients with traumatic brain injury, SNS activation induces IL-10 release, which is associated with an immunosuppressive monocyte phenotype and increased infection rate. Norepinephrine, as well as epinephrine, was found to exert immunosuppressive effects in LPS-stimulated human whole blood, as well as isolated monocytes *in vitro* (15). A study of monocytes from congestive heart failure patients again demonstrated norepinephrine’s immunomodulatory effects through β‐ADRs, with notable attenuation of IL-10 production in CHF patients (20).

Studies are limited regarding norepinephrine’s effects on granulocytes. While limited clinical data exists regarding specific immunologic effects of norepinephrine in humans, observational studies have shown increased mortality with elevated arterial norepinephrine levels (15, 21). NE has also been shown to directly promote both gram-positive and gram-negative bacterial growth *in-vitro* (15). Other studies have shown that NE reduced polymorphonuclear (PMN) cell migration, CD11b/CD18 expression, and production in response to stress (22). Beis et. al., found that NE signaling resulted in increased neutrophil and monocyte numbers during psychosocial stressors which could be reduced by blockade of β‐ADRs (23).

**Figure 1:**
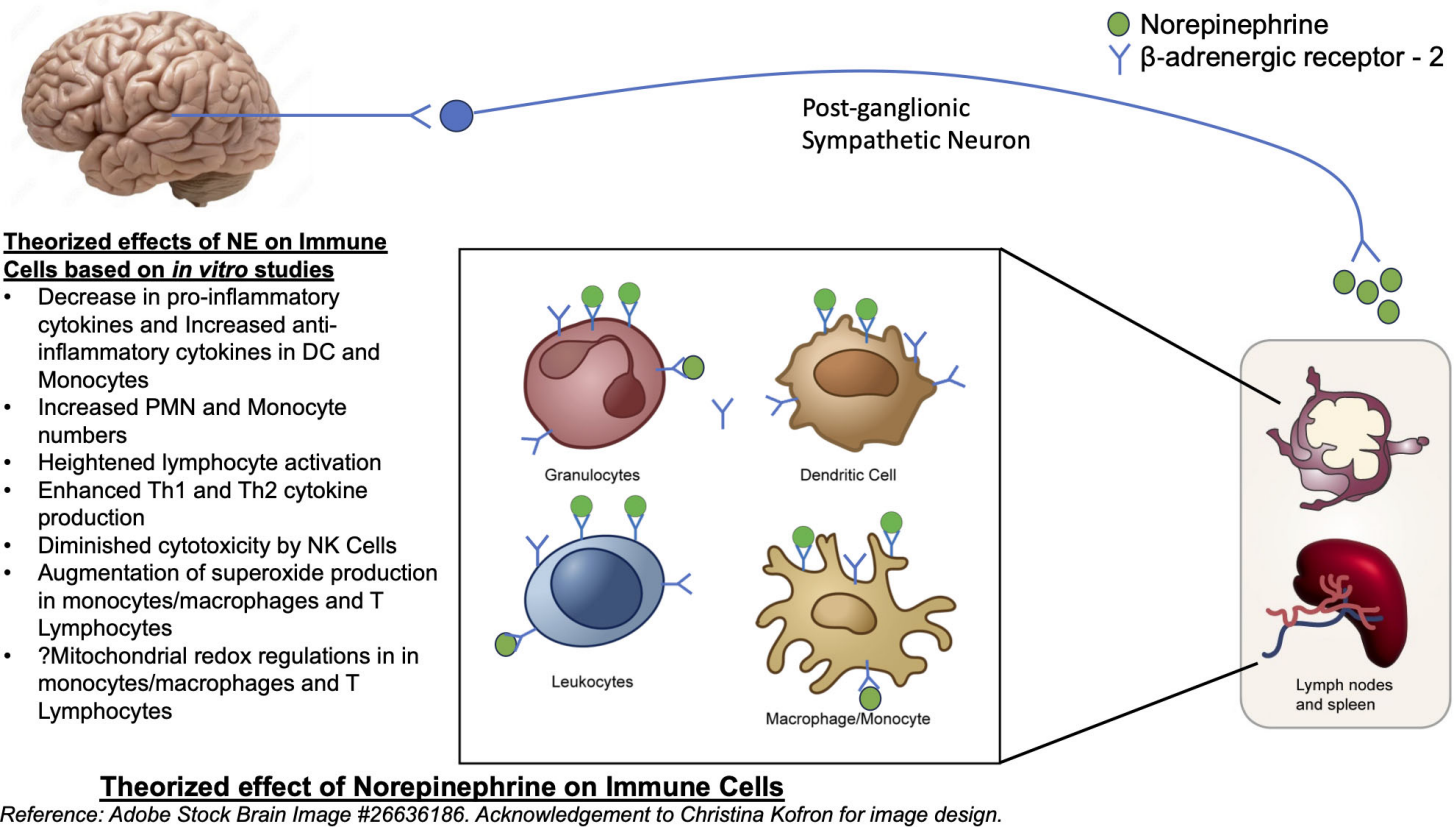
Theorized effect of Norepinephrine on Immune cells. Reference: Adobe Stock Brain Image #26636186.

NE also generates diverse regulatory patterns in T-lymphocytes (24). Many studies have demonstrated that catecholamines prompt heightened lymphocyte activation coupled with intense Th1 and Th2 cytokine production. A significant portion of these effects are orchestrated by β‐ADRs (25). Gene expression analysis of *in-vivo* memory CD8 T cells, which express higher levels of beta-adrenergic receptors compared to naïve cells, has shown to have increased expression of inflammatory cytokines (26). Adrenergic signaling has increased expansion and function of natural killer cells *in vivo*, in response to viral infection (27). Administration of norepinephrine causes an early transient elevation in overall number and function of CD8^+^ T cells and NK cells, though did not show significant changes in CD4^+^ T cells or B cells (28). **Figure 1** summarizes the theorized effects of NE on immune cells.

## Oxidative metabolism and sepsis

Reactive oxygenated species (ROS) are small short-lived oxygen-containing molecules that are highly reactive due to the negative charge accumulated by an excess oxygen molecule. Examples of ROS include superoxide anions, hydrogen peroxide (H_2_O_2_), and hydroxyl radicals. ROS are byproducts of reactions occurring in various cellular compartments i.e., the cytoplasm, cell membrane, endoplasmic reticulum (ER), mitochondria and peroxisome, as integral processes of fundamental metabolic function. ROS participates in normal physiological processes of the immune system. ROS signaling plays a significant role in immune cell activation, differentiation and signaling (9, 29–31). It has been reported that ROS participates extensively in T cell activation (9, 29–32). In macrophages and other phagocytic immune cell lines ROS play a significant role in activation and in their microbicidal activity (9, 29–31). The major source and generation of intracellular ROS is the mitochondrial electron transport chain. Under basal conditions there is a balance between oxidative and anti-oxidative metabolism which maintains mitochondrial homeostasis (29, 30).

ROS assume a crucial role in both antimicrobial host defense and inflammation. In humans, their deficiency leads to recurrent and severe bacterial infections, whereas uncontrolled release results in excessive inflammation (33). Upon activation of surface receptors, host immune cells release substantial amounts of ROS at infection sites (33). Simultaneously, activation by Fc and integrin directly induces heightened ROS production (33). Moreover, G-protein coupled receptors (GPCRs) binding to bacterial peptides can both prime cells and trigger low levels of ROS production (33). The engagement of these receptors initiates intracellular signaling pathways, that culminate in the activation of downstream effector proteins. This process includes the assembly of the NADPH oxidase complex, ultimately leading to ROS production by this complex (33). Furthermore, ROS can permeate the membranes of bacterial pathogens and inflict damage intracellularly (33). Sustained infectious insults as seen in sepsis pathophysiology results in accelerated production and release of ROS. This accelerated production leads to inefficiencies within the mitochondria due to alterations in oxygen consumption, impaired glucose and lipid metabolism, resulting in mitochondrial dysfunction (34). Likewise, increased release of ROS leads to cellular damage, DNA damage and apoptosis in adjacent tissues and cells (35). Furthermore, persistent infection results in overwhelming and depleting the natural antioxidant defense mechanisms the body has evolved to combat excess ROS production.

There is an abundance of evidence that points to excess ROS contributing to the maladaptive responses in inflammatory states leading to metabolic and global dysfunction (35). In response to inflammatory triggers and ROS signaling, leukocytes initiate the synthesis and release of proinflammatory cytokines. Specifically, there is evidence to suggest that ROS plays a role in the release of cytokines induced by LPS, NF-κB activation by thrombin and downstream endothelial cell activation (35). Similarly, research demonstrates that mitochondrial production of H_2_O_2_ contributes to NF-κB activation in endothelial cells of aged rat arteries, and that curtailing of ROS activity can reduce hypoxia-triggered endothelial NF-κB activation and IL-6 secretion (35). Much research has also highlighted that cardiac myocyte dysfunction in sepsis is a result of continued oxidative stress (30, 36–39). Sepsis induced oxidative stress is a complex interplay of inflammatory responses, mitochondrial dysfunction and depletion of antioxidant resources. Research is still ongoing but, efforts to promote anti-inflammation and antioxidant treatments in the management of sepsis have largely been unsuccessful.

## Oxidative stress, NE and sepsis

Sepsis is characterized by widespread alterations in metabolism, both at the systemic and organ-specific level in response to an infection. These changes can escalate and lead to dysregulation which results in circulatory changes and septic shock (34, 40, 41). The most common consequences of sepsis are impaired vascular permeability, cardiac malfunction, and mitochondrial dysfunction leading to impaired metabolism and, if left unchecked, shock. It has been reported that patients treated with a non-lethal dose of endotoxin and without demonstrable organ dysfunction have enhanced metabolic rates and oxygen consumption by approximately 37-55% as to basal metabolism (42). However, patients with sepsis or septic shock do not seem to have the same enhancement of metabolism and consumption and that the level of attenuation of this response correlates with disease severity (42). Precise mechanisms responsible for this attenuation is unknown (30).

NE has been identified as being superior to other vasopressors in a systematic review of randomized controlled trials evaluating in-hospital and 28-day mortality in septic shock patients (43). A meta-analysis showed that early administration of NE, which varied per study evaluated but was defined <6 hours after identification of septic shock, resulted in statistically significant reduction in short-term morality (44). The mechanisms for this remain unclear, but NE plays a role in regulating the immune response to infection and combating shock.

There is also evidence to suggest that NE is intimately involved in regulating oxidative metabolism. Research has shown that NE augments the generation of superoxide in freshly isolated primary human PBMCs by means of NADPH oxidase, mediated by α-adrenergic receptors. This effect promotes adherence of monocytes to the endothelium (24, 45). Case et. al., demonstrated that mitochondrial metabolism and superoxide-mediated redox signaling play a regulatory role in the T-lymphocyte response to NE (24). They hypothesized that the T-lymphocyte phenotype could be influenced by NE and superoxide production which could result in specific changes in cytokine expression (24). Mechanisms elucidating the role of NE in lymphocyte activation and regulation remain unclear but, oxidative metabolism may be involved. In ovarian surface epithelial cells, NE is protective against bleomycin induced oxidative damage (46). Additionally, Case et al. posited that NE appears to have a multifaceted regulatory effect on various cellular processes including metabolism and mitochondrial redox regulation (24). Systemic diseases associated with elevated inflammatory signaling and metabolic dysfunction such as atherosclerosis, diabetes, COPD, vitiligo, heart failure, Parkinson’s disease and stroke have all demonstrated altered redox balances as a potential mechanism for disease progression (47–51). Dysregulation of PBMC redox balance has been implicated in many of the disease processes listed above (47–51).

## Immune cell specific dysregulation in sepsis

Immune cell specific dysregulation in sepsis is a relatively new avenue of research that has been highlighted due to pandemic research and the advent of single cell RNA sequencing technology. Three notable studies utilized scRNAseq to identify specific PBMC populations contributing to disease progression in sepsis (52–54). Wen et. al., found a distinct monocyte population, CD14+ monocytes, that were important in the early recovery phase of Sars-CoV-2 infected patients whereas clonal expansion of T and B cells were important in later recovery stages (52). Zhang et. al., found that intensive expansion of highly cytotoxic effector T cell subsets, was associated with convalescence in moderate Sars-CoV-2 patients. In severe SARS-CoV-2 patients, there was profound immune exhaustion and broad T cell expansion. This study illustrates the dynamic nature of the immune system in response to Sars-CoV-2 (53). Similar to Wen et. al, Reyes et. al, found that in emergency department patients presenting with sepsis due to urinary tract infections the same CD14+ monocytes were present in the blood of these patients (54). They noted that this monocyte population was also present in ICU patients with sterile inflammation. They hypothesized that particular gene signatures may be able to distinguish sterile *vs* non-sterile inflammatory populations (54). In this review, we have highlighted the ubiquitous effects of NE and its impact on immunity in sepsis. Therefore, it is possible that NE may mediate some of the dysfunction in specific cell populations in sepsis which may underlie sepsis pathogenesis. This research is still in its nascent stages and is ongoing.

## NE and trained immunity in sepsis

Trained immunity refers to the long-lasting memory traits of innate immunity. Specifically, studies have shown that sustained changes in epigenetic marks and metabolic pathways can leads to an altered transcriptional response to a subsequent challenge (55, 56). The implications of trained immunity are that reprogrammed immunocytes can respond more rapidly and effectively, in particularly to distinct pathogens and markers of infection allowing for improved immune function. In sepsis, however, these trained immunity programs may lead to antagonistic inflammatory cues and ultimately tolerance of an infectious burden. In contrast, tolerance may also allow for repair and protective mechanisms (57). The exact relationship is not clearly understood and there is little to date on trained immunity in sepsis (55), but its implications could lead to the development of novel treatment strategies. Zhang et. al., has identified 3 subpopulations of monocytes with distinct cellular transcriptional programs in response to induction by 4 different inducers of trained immunity (56). These findings are consistent with the studies regarding distinct monocyte populations identified in response to COVID-19 infection (52–54). There is also some literature linking catecholamines with trained immunity. Netea et. al., found that exposure to high levels of catecholamines can result in long lasting pro-inflammatory changes in myeloid cells in cardiovascular disease (55). Similarly, Slusher et. al., found that catecholamine release in acute maximal exercise can exert a pro-inflammatory response in isolated monocytes exposed to LPS (58). There is a role here for the sympathetic nervous system in trained immunity in sepsis but, it remains unclear at this time. It is clear, however, that this is a highly complex, multi-faceted event that is regulated by diverse alterations of signaling pathways, chromatin modulation and metabolic re-wiring. Further research is needed to elucidate the intricacies of nervous system input in response to infection and training of immunocytes.

## Impact of NE and β-blockers in septic shock

There is a growing body of evidence to suggest that early norepinephrine administration during sepsis resuscitation may be beneficial in mitigating shock (44, 59–61). In 2019, the phase II CENSER trial identified that early low dose norepinephrine in adults with sepsis and hypotension results in significantly increased shock control by 6 hours (60). In 2020, a prospective ICU based propensity-score based analysis also demonstrated that an early start of norepinephrine might be safe and limit fluid resuscitation and lead to better outcomes (62). However, these studies have only focused on the vascular effects and have not evaluated the impact of NE on the immune system. Stolk et. al., performed a bench to bedside study in mice and humans that found that NE has anti-inflammatory effects in sepsis and that could lead to deleterious effects and immunoparalysis which could contribute to sepsis progression (21). This study contradicts much of the clinically relevant data that demonstrates a beneficial effect of NE in sepsis treatment. Limiting Stolk et. al.’s findings are that their study did not directly assess immunoparalysis and limited their analysis on the cytokines TNF-a and IL-10 to assess host response (63).

The value of NE in the management of septic shock has always been understood to be a result of its effect on the vascular endothelium and promotion of cardiac output preventing circulatory collapse. Traditionally, this has translated to shortened hospital length of stays and reduced mortality in septic shock (64). However, some recent trials have highlighted that allowing for some permissive hypotension in patients ≥65 years with septic shock showed no differences in 90-day mortality, and higher blood pressure values (≥65 mmHg) did not add further benefits (65, 66). The Surviving Sepsis Campaign (SSC) has suggested adding vasopressin as an adjunctive therapy to NE in the management of septic shock with the intent of rising MAP to target with the thoughts that this would prevent the deleterious consequences of an excessive adrenergic load (67). However, these recommendations were weak and based on low quality of evidence. Furthermore, there haven’t been any large scale trials evaluating the efficacy of vasopressin compared to NE (68).

There is also growing evidence to suggest β-blockers may have a positive impact on mortality in sepsis. The BEAST study found that β-blockers exposure prior to the onset of sepsis, maybe associated with better outcomes (69). A systematic review and meta-analysis found that patients with persistent tachycardia despite fluid resuscitation treated with β-blockers had lower 28-d mortality (70). The potential benefits of β-blocker therapy in sepsis include improved heart rate control thereby decreasing myocardial oxygen demand in septic patients (71). In addition, β-blockers are also thought to block the adrenergic up-regulation thought to contribute to sepsis mortality (72). Similar to Stolk et. al.’s findings, if true, this would argue against NE treatment as NE’s effects are mediated by the β2 adrenergic receptor. No large scale clinical trial has evaluated the effect of β-blockers in the treatment of sepsis and the risk of hypotension has somewhat also limited their evaluation (72).

As this review as highlighted, the role of NE is quite complex. It is clear that NE has ubiquitous effects on a variety of immune cells and vascular endothelium which can impact sepsis pathogenesis. Further research is necessary to realize NE’s impact on immunity in sepsis. There must be a reason that for the past 50 years, NE remains the first-line vasopressor of choice and has the best safety and tolerance profile in patients with septic shock (63).

## Discussion

It is clear that NE has a multi-faceted role in sepsis and this review only begins to shed light on the intricate interaction between the nervous system, immune system and immunocyte oxidative metabolism. This review highlights that there are intertwined relationships between the nervous and immune systems where communications occur in a bi-directional manner. Sepsis is ranked as a top contributor to mortality in the United States and treatment paradigms have yielded little results in mitigating mortality. The scope of sepsis research has predominantly concentrated on reducing pathogen load and providing supportive care with measures like anti-pyretics, fluids, antibiotics/anti-virals and anti-inflammatory agents (73). There has been a notable shortage of emphasis on uncovering strategies for immune system augmentation. Instead of exclusively focusing on diminishing pathogen burden, perhaps treatment can also focus on immune enhancing strategies harnessing inherent responses to infection. Furthermore, elucidating the trained immune response, or identifying specific immune cell populations involved in sepsis mediated immune dysfunction will be key to the development of novel treatment strategies. NE has been associated with improved outcomes in septic shock. This has been traditionally ascribed to its role as vasopressor and its effects on peripheral vascular resistance (43) However, with this knowledge that NE interacts with immune cells directly and alters their signaling patterns and metabolic pathways, particularly those that regulate ROS production, it begs the questions of whether NE has larger role in sepsis than just promoting peripheral vascular resistance. This would have implications for earlier norepinephrine use.

This review has underscored the role of NE in governing the bodys response to infection (8–11, 13, 14, 25, 28, 74), alongside its involvement in regulating oxidative metabolism within immune cells (24, 45). The potential for NE dysregulation to contribute to sepsis pathology is worth considering with implications for early introduction of NE in septic patients as therapy. Systemic and organ-specific changes in bioenergetics and metabolism characterize the dysregulated response to infection is sepsis and septic shock. Understanding the pathophysiological mechanisms underlying SNS regulation and mitochondrial dysfunction in sepsis may pave the way for new diagnostic strategies and therapeutic approaches. These findings may help physicians to identify distinct subgroups of sepsis patients or even sub-populations of cells for more directed treatment strategies.

## Author contributions

JT: Conceptualization, Writing – original draft. PM: Writing – original draft. BB: Writing – review & editing. DS: Writing – review & editing. JF: Writing – review & editing.
